# Supplementary material for: Midbrain signaling of identity prediction errors depends on orbitofrontal cortex networks
Source: Nat Commun. 2024 Feb 24;15:1704. doi: 10.1038/s41467-024-45880-1 (PMC10894191; doi:10.1038/s41467-024-45880-1)
Supplement: Supplementary file 1 — Supplementary Information [file 41467_2024_45880_MOESM1_ESM.pdf]

Supplementary Information for

Midbrain signaling of identity prediction errors depends on orbitofrontal cortex networks

Qingfang Liu[1], Yao Zhao[1], Sumedha Attanti[2], Joel L. Voss[3], Geoffrey Schoenbaum[1], Thorsten Kahnt[1]*

[1]*National Institute on Drug Abuse Intramural Research Program, Baltimore, MD, 21224, USA*
[2]*Mayo Clinic Alix School of Medicine, Scottsdale, AZ, 85259, USA*
[3]*Department of Neurology, The University of Chicago, Chicago, IL, 60611, USA*

*Corresponding author

Inventory of Supplementary Information

- **Supplementary Note**
- **Supplementary Fig 1**
- **Supplementary Fig 2**
- **Supplementary Fig 3**
- **Supplementary Fig 4**
- **Supplementary Fig 5**
- **Supplementary Fig 6**
- **Supplementary Table 1**
- **Supplementary References**

Supplementary Note

Control analyses accounting for discomfort and perceived TMS intensity

Despite our attempts to obscure the somatosensory differences between the sham and cTBS sessions, the cTBS session was rated as more uncomfortable and more intense than the sham session. This poses a potential confounding factor, as the observed cTBS effect may be caused by subjects' discomfort and perceived TMS intensity rather than the intended stimulation effect on the brain. To rule out this possibility, we included the self-reported discomfort and perceived TMS intensity as predictors into the linear mixed effect models for each analysis presented in **Fig 2c, 4b, 5c, 6d**, and tested whether adding TMS as a predictor significantly improved the model fit (after accounting for the effects of the two control variables). Specifically, we compared the model fits of two linear mixed effect models: a full model that included the effects of subjects, TMS session, discomfort, and intensity as predictors, and a reduced model without the TMS session effect. Additionally, for the voxel-wise global connectedness analysis, we added the subject-wise difference (sham - cTBS) between the two TMS ratings as a covariate in the group-level one-sample t-test.

In brief, all these analyses replicated our original results, demonstrating that discomfort and perceived TMS intensity cannot account for our results. The results of these analyses are described in more detail below.

- To test if changes in the voxel-wise global connectedness were driven by subjects' physical experience, we included discomfort and perceived TMS intensity differences (sham – cTBS) as covariates in the one-sample t-test, which yielded almost identical maps as those shown in **Fig 2a, b**.
- To test if the TMS effects on global connectedness in the OFC and LPFC ROIs remained significant after accounting for potential confounding factors of self-reported discomfort and perceived TMS intensity, we compared the model fits of two linear mixed effect models on predicting global connectedness for both the OFC (focused on time bins 1 and 2) and LPFC (focused on time bins 2, 3, and 4) effects. Adding TMS session as a predictor significantly improved the model fit for most time bins (all p's <0.05), except for the 3rd time bin in the LPFC ($p = 0.183$).
- To test if TMS modulated the change in accuracy from pre-reversal to post-reversal after accounting for potential confounding factors of self-reported discomfort and perceived TMS intensity, we compared the fits of two linear mixed effect models. Adding TMS session as a predictor significantly improved the model fit ($p = 0.011$).
- To test if TMS modulated the iPE-related fMRI responses after accounting for potential confounding factors of self-reported discomfort and perceived TMS intensity, we compared the fits of two linear mixed effect models that predict the change of iPE signals from reversal to post-reversal trials. This analysis was conducted separately for each ROI (midbrain, LPFC, and left OFC). Adding TMS session as a predictor significantly improved the model fit in all ROIs (all p's < 0.05).
- To test if the effects of TMS on neural representations of expected reward identity in the lateral OFC persisted after accounting for potential confounding effects of self-reported discomfort and perceived TMS intensity, we compared model fits of two linear mixed effect models. Adding TMS session as a predictor significantly improved the model fit for the effect in the lateral OFC ($p = 0.043$).

Taken together, we conclude that our results are not driven by discomfort and perceived TMS intensity.

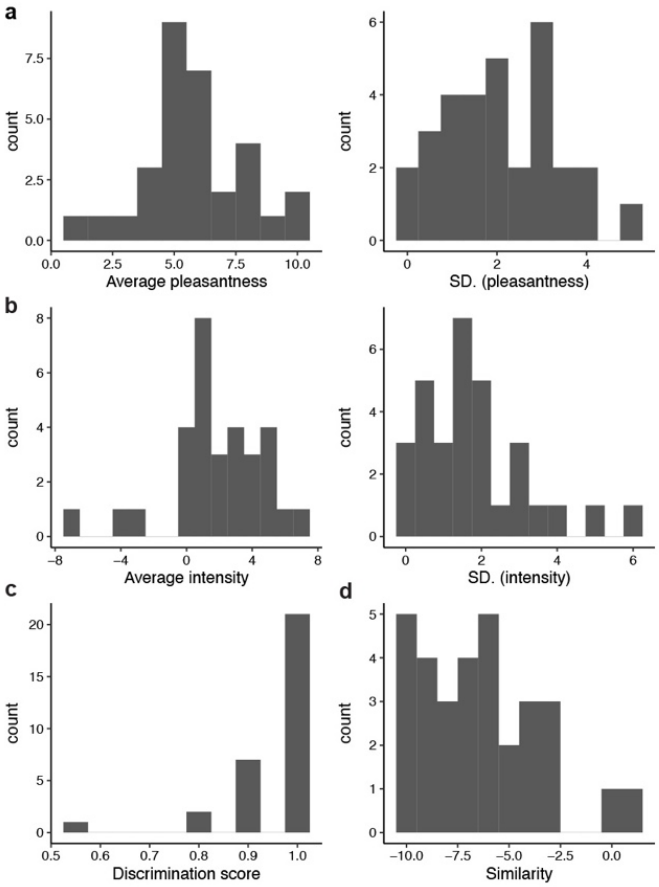

Supplementary Fig 1: Odor ratings and tests from the Screening session (Day 1, n = 31). **(a)** Distribution of mean and standard deviation of odor pleasantness ratings across subjects. For each subject, the mean and standard deviation of pleasantness ratings were calculated across the three selected odors. Pleasantness ratings range from -10 to 10, with 10 indicating the highest pleasantness. (**b)** Mean and standard deviation of odor intensity ratings across subjects. Intensity ratings range from -10 to 10, with 10 indicating the strongest intensity. (**c)** Distribution of discrimination scores across subjects. (**d)** Distribution of odor similarity ratings across subjects. Similarity ratings range from -10 to 10, with 10 indicating identical odors.

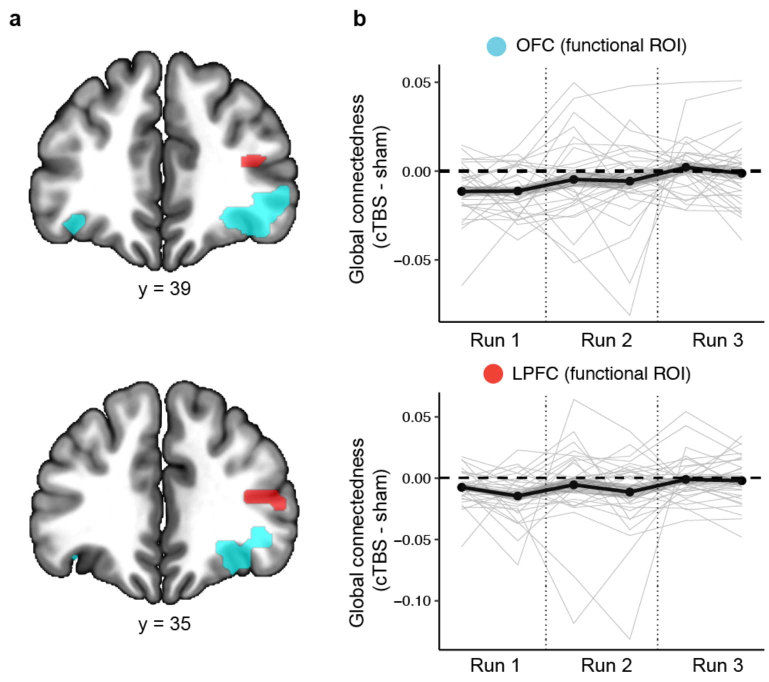

Supplementary Fig 2: Global connectedness (cTBS – sham) in functional ROIs of OFC and LPFC. (a) Functional ROIs in OFC and LPFC that are defined based on the voxel-wise comparison between sham and cTBS, shown in **Fig 2a, b** (thresholded at $p < 0.001$, uncorrected). **(b)** Global connectedness difference (cTBS - sham) within the two functional ROIs. Note that these ROIs were defined based on the effect of TMS, and thus plots are purely illustrative. Bold black line depicts the mean and shaded area the standard error across subjects ($n = 31$). Thin gray lines are data from individual subjects.

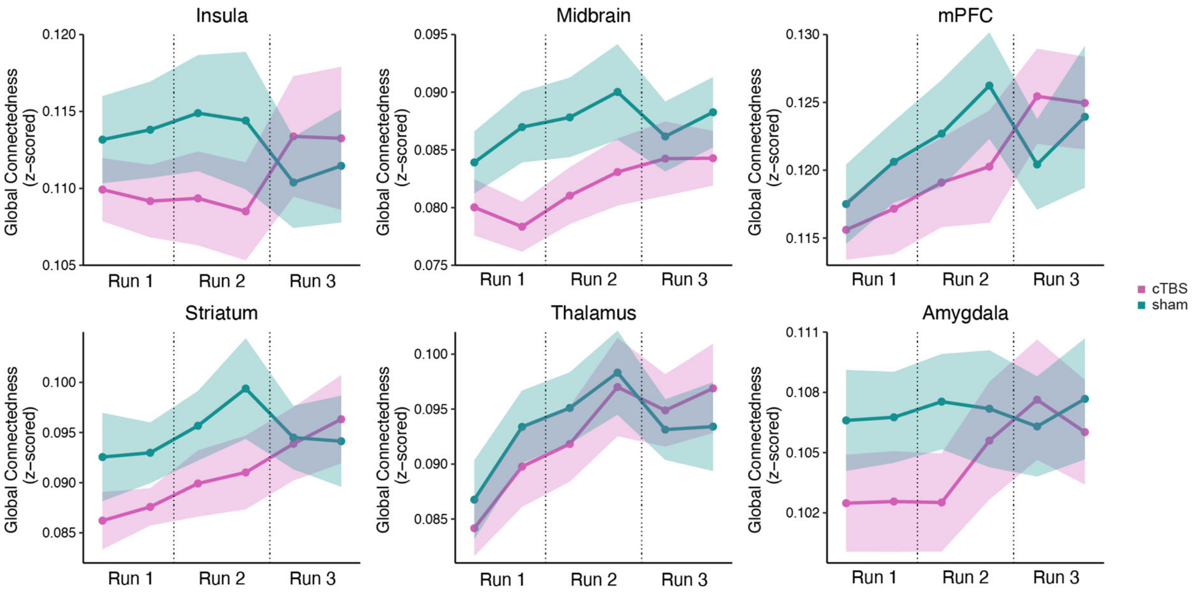

Supplementary Fig 3: Global connectedness in additional brain areas. We explored the effects of TMS on global connectedness in additional brain regions involved in value updating, namely: insula, midbrain, mPFC (including ACC), striatum, thalamus, and amygdala. These ROIs were defined based on our iPE analysis (insula, midbrain, mPFC, striatum, and thalamus) or based on coordinates identified previously[1] (amygdala). n = 31 subjects. Two-way repeated measure ANOVAs with TMS and time as factors for each area showed a main effect of TMS in the midbrain ($F(1, 30) = 5.693$, $p = 0.024$), and a main effect of time in the thalamus ($F(3.07, 92.22) = 5.004$, $p = 0.003$). However, these main effects were not qualified by significant interactions. No main or interaction effects were identified in any of the other four areas (all p's > 0.05).

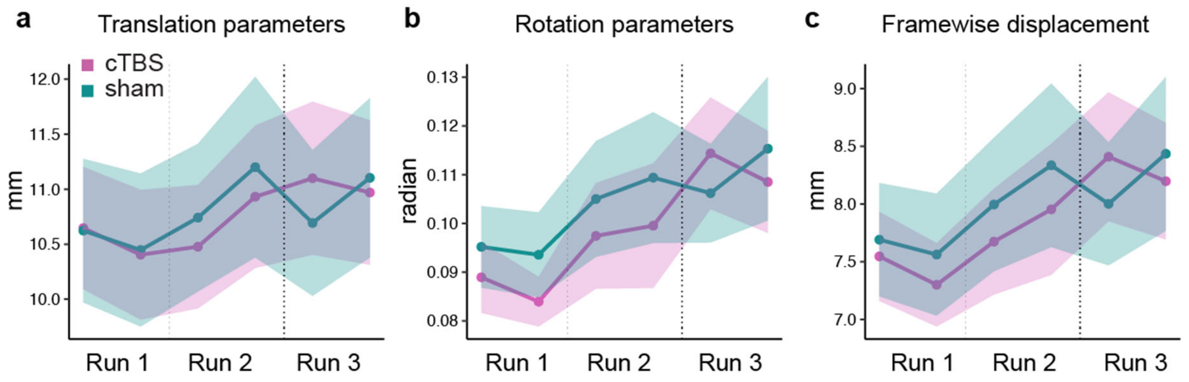

Supplementary Fig 4: Comparison of head motion parameters across runs between cTBS and sham sessions. (a) Translation parameters, **(b)** rotation parameters, and **(c)** framewise displacement during scanning. A two-way ANOVA with repeated measures on translation parameters or framewise displacement did not reveal significant main effects of TMS or time, or their interaction (all p's > 0.05). A two-way ANOVA with repeated measures on rotation parameters revealed a significant main effect of time (p = 0.01), but no main effect of TMS and no time by TMS interaction (all p's > 0.05). Additional analyses show that effects of TMS on global connectedness in OFC and LPFC (**Fig 2**) remain significant when correcting for head motion parameters. For **a, b, c**, n = 31.

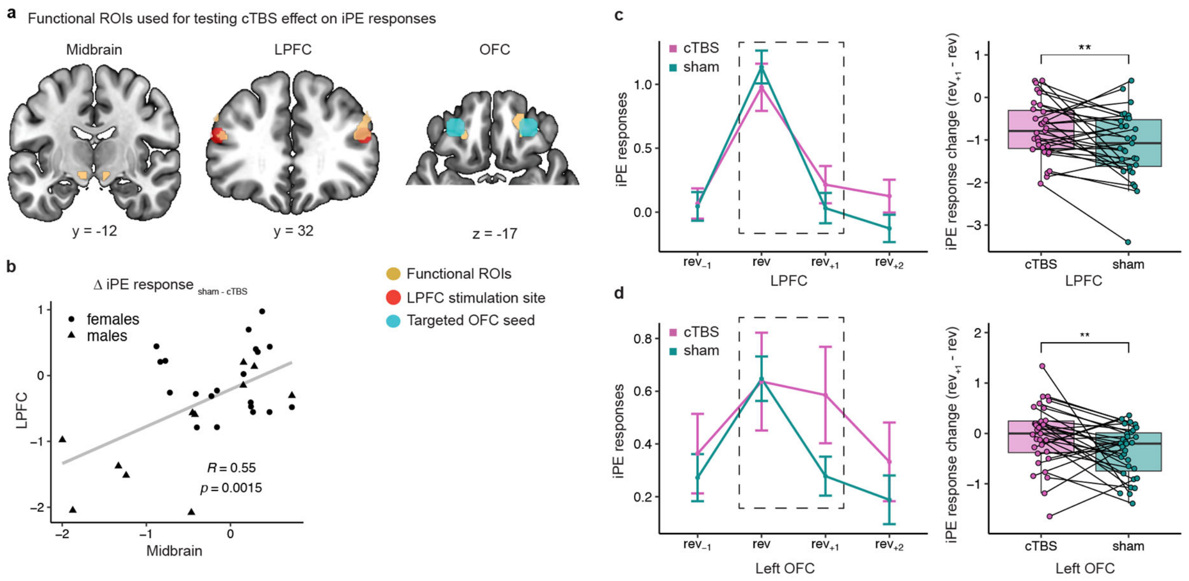

Supplementary Fig 5: Functional ROIs used for testing cTBS effect on iPEs, and additional results on iPEs. (a) Functional ROIs of the midbrain, LPFC, and OFC. Peak coordinates used for to localize clusters: left midbrain ([-10, -24, -10]), right midbrain ([10, -14, -10]), left LPFC ([-40, 6, 40]), and right LPFC ([46, 22, 30]). All voxels in the midbrain and LPFC ROIs survive whole brain FWE correction at p_{FWE} = 0.05. The red and cyan spheres denote the LPFC stimulation site and targeted OFC seed, respectively. The OFC ROI included voxels (p < 0.001, uncorrected) adjacent to the peak coordinates within the OFC (left: [-22, 30, -14], right: [20, 42, -16]). (**b)** Scatter plot showing the inter-regional relationship between the cTBS effect on iPE signals in the midbrain (x-axis) and LPFC (y-axis). The ΔiPE response was the difference between the rev_{+1} trials and the rev trials. The shape of the markers denotes the sex of the subject (dot: females; triangle: males). The gray line denotes the best linear fit of the inter-regional relationship. R values are Pearson's correlation. **(c)** Similar to Fig 5b,c, plotted for the LPFC ROI. The left panel shows the outcome-related fMRI responses in cTBS and sham sessions across rev_{-1}, rev, rev_{+1}, and rev_{+2} trials. The dashed squares indicate the change of fMRI responses from rev to rev_{+1} used in the right panel. Each pair of dots connected by a line represents one subject. The difference between TMS sessions was tested using one-sided paired t-tests. ** denotes p < 0.01. **(d)** Same as (**c**) but for the left OFC. For **b, c, d**, n = 31.

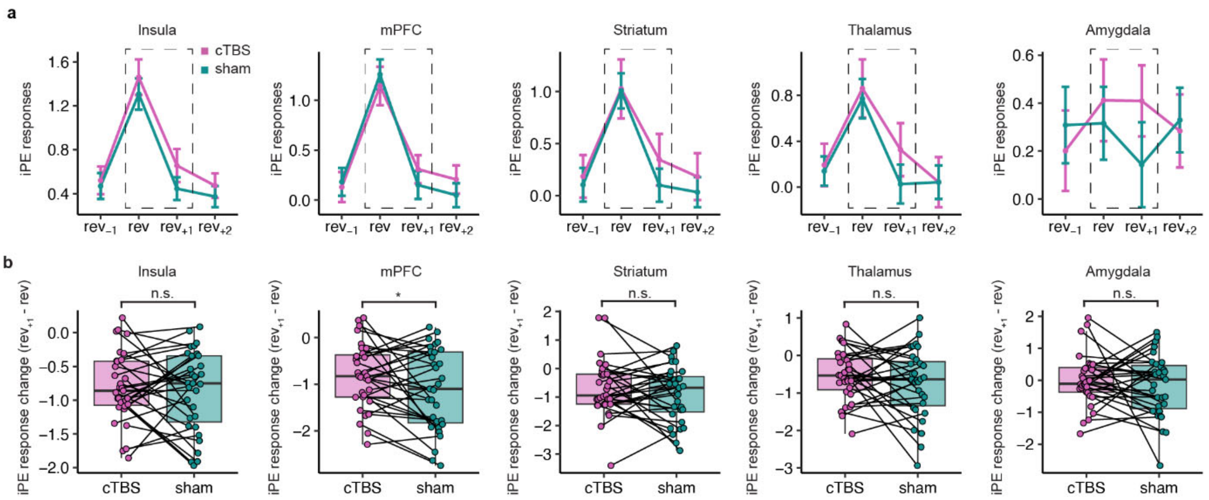

Supplementary Fig 6: Effects of TMS on iPE-related activity in additional brain areas. (a) Outcome-related fMRI responses on trials before (rev_{-1}), during (rev) and following a reversal (rev_{+1} and rev_{+2}). **(b)** Change from rev to rev_{+1} in the sham and cTBS session. ROIs were defined as described in **Supplementary Fig 3**. Note, amygdala responses did not display the typical iPE pattern because this area was not functionally defined in the same way as the other areas. * denotes $p < 0.05$ and n.s. denotes $p > 0.05$. For both **a, b**, n = 31.

Supplementary Table 1: Peak activation coordinates of the reversal effect (reversal > non-reversal, across Day 3 and Day 4 sessions, threshold at $p_{FWE} < 0.05$, k >= 25 voxels)

Brain region	x	y	z	t	p_{FWE}
Left insula	-30	20	2	12.62	9.49E-09
Right insula	38	20	-4	10.35	1.24E-06
Right insula	50	22	8	8.39	1.40E-04
Medial frontal gyrus	-2	26	50	10.05	2.46E-06
Left midbrain	-10	-24	-10	9.57	7.58E-06
Left midbrain	-10	-14	-6	8.51	1.02E-04
Right midbrain	10	-14	-10	7.98	3.52E-04
Right midbrain	8	-10	-2	8.70	6.37E-05
Right midbrain	10	-16	12	6.16	2.26E-02
Right midbrain	6	-26	-6	7.85	4.72E-04
Left striatum	-12	4	8	7.39	1.33E-03
Left striatum	-14	4	-2	6.44	1.19E-02
Right striatum	14	4	12	7.03	3.07E-03
Right striatum	16	4	0	6.90	4.10E-03
Right superior frontal gyrus	40	14	56	9.38	1.22E-05
Left lateral prefrontal cortex	-40	6	40	9.29	1.50E-05
Left lateral prefrontal cortex	-50	18	26	8.68	6.65E-05
Left lateral prefrontal cortex	-42	24	32	8.11	2.63E-04
Right lateral prefrontal cortex	46	22	30	8.29	1.78E-04
Right middle temporal gyrus	54	-24	-12	8.90	3.88E-05
Posterior cingulate	0	-34	32	7.01	3.17E-03

Supplementary References

1. Howard JD, Kahnt T. Identity prediction errors in the human midbrain update reward-identity expectations in the orbitofrontal cortex. *Nat Commun* **9**, 1611 (2018).
