# Supplementary material for: Midbrain signaling of identity prediction errors depends on orbitofrontal cortex networks
Source: Nat Commun. 2024 Feb 24;15:1704. doi: 10.1038/s41467-024-45880-1 (PMC10894191; doi:10.1038/s41467-024-45880-1)
Supplement: Supplementary file 3 — Reporting Summary [file 41467_2024_45880_MOESM3_ESM.pdf]

nature portfolio

Corresponding author(s): Thorsten Kahnt

Last updated by author(s): 1/22/2024

Reporting Summary

Nature Portfolio wishes to improve the reproducibility of the work that we publish. This form provides structure for consistency and transparency in reporting. For further information on Nature Portfolio policies, see our Editorial Policies and the Editorial Policy Checklist.

Statistics

For all statistical analyses, confirm that the following items are present in the figure legend, table legend, main text, or Methods section.

n/a	Confirmed	
☐	☒	The exact sample size (*n*) for each experimental group/condition, given as a discrete number and unit of measurement
☐	☒	A statement on whether measurements were taken from distinct samples or whether the same sample was measured repeatedly
☐	☒	The statistical test(s) used AND whether they are one- or two-sided<br>*Only common tests should be described solely by name; describe more complex techniques in the Methods section.*
☐	☒	A description of all covariates tested
☐	☒	A description of any assumptions or corrections, such as tests of normality and adjustment for multiple comparisons
☐	☒	A full description of the statistical parameters including central tendency (e.g. means) or other basic estimates (e.g. regression coefficient) AND variation (e.g. standard deviation) or associated estimates of uncertainty (e.g. confidence intervals)
☐	☒	For null hypothesis testing, the test statistic (e.g. *F*, *t*, *r*) with confidence intervals, effect sizes, degrees of freedom and *P* value noted<br>*Give P values as exact values whenever suitable.*
☐	☒	For Bayesian analysis, information on the choice of priors and Markov chain Monte Carlo settings
☒	☐	For hierarchical and complex designs, identification of the appropriate level for tests and full reporting of outcomes
☐	☒	Estimates of effect sizes (e.g. Cohen's *d*, Pearson's *r*), indicating how they were calculated

Our web collection on statistics for biologists contains articles on many of the points above.

Software and code

Policy information about availability of computer code

Data collection	The task (with visual and olfactory stimuli) was presented and behavioral data were acquired using the Cogent 2000 toolbox (v1.32) in Matlab (R2016b).<br>The breathing data were acquired using the PowerLab (ADInstruments, Dunedin, New Zealand)<br>For TMS, we used MagVenture neuronavigation software to navigate the coil.
Data analysis	The fMRI data were processed and analyzed using Statistical Parametric Mapping (SPM12) and custom code in Matlab (version R2020b, Mathworks Inc).<br>Custom code for reproducing the findings reported in this manuscript is available on GitHub (https://github.com/QingfangLiu/OFC_midbrain_TMS) with relevant instructions.<br>For figures of the whole-brain fMRI group effects, we used MRIcroGL (version 12.6) software.<br>For Bayesian model fitting to the behavioral data, we used R2jags package based on R (4.1.2)/ RStudio (2022.07.1).<br>For the glass brain plot for network-targeted TMS visualization, we used BrainNet Viewer software (Xia et al., 2013, Plos One).<br>For other analyses and visualizations of behavioral data and ROI-based fMRI data, we used packages dplyr (1.0.8), plyr (1.8.6), and ggplot2 (3.4.1) based on R (4.1.2)/ RStudio (2022.07.1).

For manuscripts utilizing custom algorithms or software that are central to the research but not yet described in published literature, software must be made available to editors and reviewers. We strongly encourage code deposition in a community repository (e.g. GitHub). See the Nature Portfolio guidelines for submitting code & software for further information.

Data

Policy information about availability of data

All manuscripts must include a data availability statement. This statement should provide the following information, where applicable:

- Accession codes, unique identifiers, or web links for publicly available datasets
- A description of any restrictions on data availability
- For clinical datasets or third party data, please ensure that the statement adheres to our policy

The data supporting the findings presented here are available on GitHub (https://github.com/QingfangLiu/OFC_midbrain_TMS). Source data are provided with this paper. Statistical group-level maps are available on NeuroVault (https://neurovault.org/collections/15898/). Midbrain probability atlas used in this study are available from the original authors (https://www.adcocklab.org/neuroimaging-tools).

Research involving human participants, their data, or biological material

Policy information about studies with human participants or human data. See also policy information about sex, gender (identity/presentation), and sexual orientation and race, ethnicity and racism.

Reporting on sex and gender	Participants' sex was determined based on self-reporting. We recruited 17 males among 42 participants. 35 out of 42 completed all sessions. After exclusion of four subjects, our participants consisted of 11 males among 31 participants.<br><br>In our within-subject design, the order of sham and cTBS was counterbalanced across subjects. When allocating subjects to different orders, we considered participants' sex such that both sexes are assigned to each order in roughly equal proportions: 17 participants out of 31 (5 males out of 11, 12 females out of 20) received sham first.<br><br>Although sex differences were not hypothesized for the present research question, we conducted sex-based analyses, testing if the cTBS effect on iPE signals or on the reward identity expectations may be modulated by subjects' sex. We only found sex-related effect on iPE signals in the LPFC ROI, but not in the midbrain ROI that we were primarily interested in. We reported sex-based analyses and results in the Method. As our main experimental conclusions were based on the midbrain effect, we believe that our results could be stated for each sex group.
Reporting on race, ethnicity, or other socially relevant groupings	We collected self-reported demographic information from each participant in the screening process, but we did not report such information in our manuscript.
Population characteristics	The age distribution of the reported 31 subjects (after excluding four subjects from 35 completed subjects): 19 - 42, mean = 26.39, SD = 6.04. For imaging analyses, we conducted Pearson's correlations testing the relationship between the cTBS effect (on iPE signals or on the reward identity expectations) and subjects' age. None of the analyses suggested a significant age-related effect or such a trend. We reported these results in the Method.
Recruitment	Research participants were students or employees based in Chicago, IL, and were recruited through advertisement (convenience sampling). It is very unlikely though that the study of basic brain learning function would be biased by the recruitment procedure.
Ethics oversight	The experiment protocol was approved by the Northwestern University Institutional Review Board. Written informed consent was obtained from each participant prior to the experiment. We included this information in the manuscript.

Note that full information on the approval of the study protocol must also be provided in the manuscript.

Field-specific reporting

Please select the one below that is the best fit for your research. If you are not sure, read the appropriate sections before making your selection.

☒ Life sciences ☐ Behavioural & social sciences ☐ Ecological, evolutionary & environmental sciences

For a reference copy of the document with all sections, see nature.com/documents/nr-reporting-summary-flat.pdf

Life sciences study design

All studies must disclose on these points even when the disclosure is negative.

Sample size	No explicit power calculations were performed to determine sample sizes. The total number of subjects were determined by the limits of data collection within a reasonable time, and were comparable to similar TMS or TMS-fMRI studies.
Data exclusions	We excluded four subjects from all analyses (resulting in 31 remaining subjects): two because they did not tolerate cTBS at 80% rMT and two due to poor odor identification performance (described in Methods).
Replication	We did not replicate these results in a separate cohort of subjects with the constraints of the study timeline and available resources.
Randomization	We randomly allocated research participants into two groups, each group receiving sham and cTBS in different orders (sham first or cTBS first, hence within-subject design). The randomization was determined by pre-generated binary random numbers in Matlab. When allocating

	subjects to different orders, we also considered participants' sex such that both sexes were assigned to each order with roughly equal proportion: 17 participants out of 31 (5 males out of 11, 12 females out of 20) received sham first.<br>We also conducted independent t-tests to examine if TMS order modulated the TMS effect on iPE and reward identity expectations, but we did not find any significant effects or trends.
Blinding	The TMS operator was not blinded to group allocation. The two conditions (cTBS or sham) were altered by using the appropriate side of the Cool-B65 A/P (Active/Placebo) coil to minimize differences between active and sham TMS.<br>The MRI technicians were blinded to group allocation, who talked to participants during the task-based fMRI.<br>During data collection, on each session, we provided each participant streamlined and identical instructions and the same behavioral task was used in both sessions.<br>Participants were blinded to group allocation (order of active/sham TMS). We conducted survey after each session and after the experiment to assess their knowledge about group allocation (described in Methods).<br>During data analysis, we performed identical analysis on participants from each group and we do not anticipate any bias.

Reporting for specific materials, systems and methods

We require information from authors about some types of materials, experimental systems and methods used in many studies. Here, indicate whether each material, system or method listed is relevant to your study. If you are not sure if a list item applies to your research, read the appropriate section before selecting a response.

Materials & experimental systems

n/a	Involved in the study	
☒	☐	Antibodies
☒	☐	Eukaryotic cell lines
☒	☐	Palaeontology and archaeology
☒	☐	Animals and other organisms
☒	☐	Clinical data
☒	☐	Dual use research of concern
☒	☐	Plants

Methods

n/a	Involved in the study	
☒	☐	ChIP-seq
☒	☐	Flow cytometry
☐	☒	MRI-based neuroimaging

Plants

Seed stocks	*Report on the source of all seed stocks or other plant material used. If applicable, state the seed stock centre and catalogue number. If plant specimens were collected from the field, describe the collection location, date and sampling procedures.*
Novel plant genotypes	*Describe the methods by which all novel plant genotypes were produced. This includes those generated by transgenic approaches, gene editing, chemical/radiation-based mutagenesis and hybridization. For transgenic lines, describe the transformation method, the number of independent lines analyzed and the generation upon which experiments were performed. For gene-edited lines, describe the editor used, the endogenous sequence targeted for editing, the targeting guide RNA sequence (if applicable) and how the editor was applied.*
Authentication	*Describe any authentication procedures for each seed stock used or novel genotype generated. Describe any experiments used to assess the effect of a mutation and, where applicable, how potential secondary effects (e.g. second site T-DNA insertions, mosiacism, off-target gene editing) were examined.*

Magnetic resonance imaging

Experimental design

Design type	We used both event-related task-based MRI (Day 3 and Day 4) and resting-state MRI (Day 2).
Design specifications	Participants performed three runs of task on each of the Day 3 and Day 4, immediately following the TMS.<br>Each run involved 64 trials, lasted approximately 14.4 minutes and consisted of 430 EPI volumes.<br>Each trial lasted 10 seconds with pseudorandomized ITI between 2-4 seconds.
Behavioral performance measures	We recorded which button was pressed and the response time on each trial. We calculated mean and standard deviation to characterize behavioral performance.

Acquisition

Imaging type(s)	Functional and structural images
Field strength	3 Tesla
Sequence & imaging parameters	MRI data were acquired on a Siemens 3T PRISMA system equipped with a 64-channel head-neck coil.

For resting-state fMRI on Day 2, 250 echo-planar imaging (EPI) volumes were acquired with a parallel imaging sequence with the following parameters: repetition time, 2 s; echo time, 22 ms; flip angle, 80°; multi-band acceleration factor, 2; slice thickness, 2 mm, no gap; number of slices, 58; interleaved slice acquisition order; matrix size, 104 ´ 96 voxels; field of view, 208 mm x 192 mm. The functional scanning window was tilted ~30° from axial to minimize susceptibility artifacts in the OFC.

For structural images, we acquired 1mm isotropic T1-weighted structural scan using a MPRAGE sequence.

On Day 3 and Day 4, the task-based fMRI data were acquired with the same parameters as the resting-state scan.

To aid coregistration between the functional scans and the anatomical image, ten whole-brain EPI volumes were acquired for each subject on Day 2, Day 3, and Day 4, using the same scanning parameters as the resting-state scan except covering the whole brain (95 slices) with a repetition time of 3.15 s.

Area of acquisition

A whole-brain scan was used.

Diffusion MRI ☐ Used ☒ Not used

Preprocessing

Preprocessing software

Statistical Parametric Mapping (SPM12) software. We used Gaussian smoothing kernel of 6 mm full-width half maximum (FWHM) in all three spatial dimensions. Only for multivariate imaging data analysis, we used Gaussian smoothing kernel with size of 2mm FWHM in all three spatial dimensions.

Normalization

For spatial normalization, the T1 anatomical image from each subject was normalized to the Montreal Neurological Institute (MNI) space using the six-tissue probability map provided by SPM12, and the resulting deformation fields were applied to the functional EPIs to transform them into MNI space. Affine transforms (that include displacement, rotation, reflection, zooming, and shearing) were employed by SPM in this process, thereby causing nonlinear transformations.

Normalization template

MNI space (in SPM12)

Noise and artifact removal

During imaging data preprocessing, for each subject, we corrected for head movement by realigning all functional EPIs from all runs and sessions to the first acquired image.

We measured subjects' sniffing directly at the nasal mask using a respiratory flow head and a spirometer, and recorded using PowerLab equipment (ADInstruments, Dunedin, New Zealand) at a sampling rate of 1 kHz. Individual sniffing traces for each fMRI run were smoothed with a 250ms moving window, down-sampled to 10 Hz, high-pass filtered (50s cutoff) to eliminate slow signal drifts, normalized by subtracting the mean and dividing by the standard deviation across the run trace, and finally down-sampled to 0.5 Hz to align with the scanning TR. Subjects' nasal airflow was characterized by these traces, and sniff volumes were calculated as the integral of the nasal airflow.

We included the following nuisance regressors in all fMRI analyses: the smoothed, normalized sniff trace, down-sampled to scanner resolution (0.5 Hz) indicating nasal airflow, sniff volume, and the squares of both sniff measures; the six realignment parameters (three translations, three rotations) calculated for each volume during motion-correction; the derivative, square, and the square of the derivative of each realignment regressor; the absolute signal difference between even and odd slices, and the signal variance across slices, in each functional volume (to account for fMRI signal fluctuation caused by within-scan head motion); the squares, derivatives, and squared derivatives of these two within-volume measures; and additional dummy regressors as needed to model out individual volumes in which particularly strong head motion occurred (see Volume censoring below).

Volume censoring

We identified volumes with excessive within-scan head motion, by calculating two quantitative metrics: the signal difference between even and odd number of slices, and signal variance across slices. Each metric was subsequently normalized and their absolute values were computed across subjects and runs. Volumes exhibiting values exceeding 4 standard deviations (STD) on either metric were categorized as "bad volumes". We used similar criteria in our previous publications (e.g., Howard & Kahnt, 2018, Nature Comm).

Across subjects and runs, 51.61% of the runs were found to contain at least one such bad volume. The number of bad volumes per run ranged from 0 to 13, highly right-skewed, with an average of 2.02 (given 430 volumes per run). Bad volumes were further included as dummy nuisance regressors to regress them out in all GLMs.

Statistical modeling & inference

Model type and settings

We conducted both mass univariate and multivariate pattern-based analyses.

Mass univariate analysis: To identify neural correlates to iPEs, we constructed subject-level event-related GLMs using regressors convolved with a canonical hemodynamic response function (HRF), and time-locked to the onset of the cue presentation and odor delivery. We modeled four different odor delivery conditions: reversal trials, one trial before, one trial after, and two trials after a reversal. To increase the statistical efficacy, the odor delivery regressors from all three runs from a given session were concatenated. The cue-locked regressors from all six runs were combined into a single regressor. As a result, each subject-level GLM contained nine task-related regressors, along with nuisance regressors (see Noise and artifact removal above). We computed single-subject contrast images, comparing fMRI responses on reversal trials with responses on non-reversal trials across both sessions. In the 2nd level analysis, we conducted one-sample t-test comparing the individual contrast images against zero.

Multivariate pattern-based similarity analysis: In a first step, we estimated single-trial beta values time-locked to the cue onsets. For each trial, we specified a GLM with the regressor of interest being the "cue" onset of the current trial, and other regressors including the cue onsets of all other trials, "odor outcome" onsets of the current trial, "odor outcome" onsets of all other trials, and nuisance regressors. We then computed the difference in correlations as a neural measure of identity expectations. The correlation coefficients were Fisher's z-transformed, subtracted, and averaged across reversals per session to obtain reward identity expectations. We conducted whole-brain searchlight analysis obtaining voxel-wise identity expectation measures for each subject. In the 2nd level analysis, we conducted one-sample t-test comparing the reward identity expectation signals against zero.

Effect(s) tested

We tested for different effects separately in the mass univariate analysis and multivariate pattern-based similarity analysis.
Mass univariate analysis: to examine neural responses to iPEs (i.e. the neural responses to odor outcomes at reversal trials, compared with non-reversal trials)
Multivariate pattern-based similarity analysis: to examine neural responses to reward identity expectations (i.e. pattern-based information on expected reward identity at cue onsets)

Specify type of analysis: ☐ Whole brain ☐ ROI-based ☒ Both

Anatomical location(s)

We used functional ROIs in each of the mass univariate and multivariate pattern-based analyses, but they were separately defined under each analysis.
In the mass univariate analysis of the iPE effects, we created three functional ROIs (midbrain, LPFC, and OFC) based on the contrast of reversal > non-reversal from both cTBS and sham sessions.
In the multivariate pattern-based analysis: we used functional ROI of lateral OFC based on identity expectation > 0, that overlapped with the targeted OFC seed regions.
Importantly, these functional ROIs were defined independent/orthogonal of the TMS effect of interest, by collapsing across data from both sessions.

Statistic type for inference

(See Eklund et al. 2016)

voxel-wise inference

Correction

When testing the iPE effect, significant voxels were identified using a threshold of $p<0.05$ corrected by a whole-brain family-wise error rate (FWER) at the voxel level. When testing the identity expectation effect, we used a threshold of $p<0.05$, corrected for multiple comparisons within the lateral OFC seed region.

Models & analysis

n/a	Involved in the study
☒	☐ Functional and/or effective connectivity
☒	☐ Graph analysis
☒	☐ Multivariate modeling or predictive analysis
